# Emotional dysregulation predicts problematic gaming in children and youths: a cross-sectional and longitudinal approach

**DOI:** 10.1007/s00787-023-02184-x

**Published:** 2023-03-18

**Authors:** Leonie Marie Schettler, Rainer Thomasius, Kerstin Paschke

**Affiliations:** German Center for Addiction Research in Childhood and Adolescence (DZSKJ), University Medical Center Hamburg-Eppendorf (UKE), Martinistraße 52, 20246 Hamburg, Germany

**Keywords:** Gaming disorder, Problematic gaming, Adolescents, Emotion regulation, Longitudinal study

## Abstract

Adolescents show a high vulnerability for addictive gaming patterns on the one hand and immature emotion regulation (ER) abilities as a risk factor for mental disorders on the other hand. We investigated the predictive value of ER difficulties on problematic gaming (PG) considering age groups (children vs. youths) and gender cross-sectionally and prospectively in a representative sample of German adolescents via online survey with two measurement points 14 months apart. General Poisson, logistic, and multinomial regression models were estimated to predict gaming patterns by ER difficulties controlling for age group and gender. Results revealed ER difficulties to be significantly associated with PG. Moreover, subgroup analyses indicated differing ER patterns for children vs. youths and boys vs. girls: for children, higher PG values were associated with emotional awareness and emotional clarity whereas for youths it was the acceptance of emotional responses. Moreover, gender differences implicated that boys with PG had more deficits in goal-oriented behavior as well as emotional awareness while affected girls were lacking emotional clarity and had problems with the acceptance of their emotional responses. Interestingly, procrastination was a significant predictor for PG irrespective of subgroups. Furthermore, longitudinal analyses indicated that difficulties in ER promoted PG while stronger procrastination tendencies maintained it. With the inclusion of procrastination, which can be understood as a maladaptive ER strategy, a broader picture of ER difficulties as a risk factor for PG could be drawn. The findings support a better understanding of PG etiology and the development of targeted prevention and intervention measures.

## Introduction

### Problematic gaming in adolescents

In the course of the technological progress of the last decade, computer, console and mobile games became a regular companion in the everyday life of many adolescents. Gaming times increased during the COVID-19 pandemic and repeated (partial) lockdowns with the closure of schools or leisure facilities [1, 2]. Most adolescents use digital games in an unproblematic recreational way, but for some, excessive gaming leads to serious consequences due to the development of an addictive behavior interfering with academic, family, and/or social life. A recent meta-analysis with an average subject age of 17.5 years indicated a global prevalence of addictive gaming around 3% [3]. The authors of this paper emphasize the prevalence to be highest in adolescents.

Problematic gaming (PG) behavior was first introduced as Internet Gaming Disorder (IGD), a “condition for further research”, in the appendix of the fifth version of the “Diagnostic and Statistical Manual for Mental Disorders” (DSM-5) in 2013 [4]. For an IGD, five out of nine diagnostic criteria based on pathological gambling and substance use disorders need to be fulfilled within the past 12 months. Moreover, the term Gaming Disorder (GD) was recently included in the eleventh version of the "International Classification of Diseases and Related Health Problems" (ICD-11) [5]. GD is described by the following criteria: (1) loss of control over gaming, (2) increasing prioritizing of gaming and (3) continued gaming despite negative consequences which have to be present for at least 12 months and lead to significant impairment in personal, educational and social life. Furthermore, to understand potentially harmful precursor GD patterns, Hazardous Gaming (HG) has been included to describe at-risk behavior. The conceptualization of PG varies between the two classification systems due to a differing weighting of symptoms as well as resulting impairments [6]. While the DSM-5 allows a broader screening on a population level, the ICD-11 has a higher specificity to differentiate between normal, at-risk and pathological gaming [6, 7]. To account for both definitions, in this paper the term PG will be used as an umbrella term for IGD and GD.

PG is based on a complex etiology and a wide range of biological, psychological, family, and other environmental risk factors have been identified [8–10]. Regarding gender, the occurrence of PG is substantially associated with being male [8, 9]. The influence of adolescent age on PG is unclear, partly because little research has been conducted among children [9]. Especially adolescents with high levels of family conflict and poorer relationships are at high-risk for PG [8, 11]. Hence, an escape into the world of gaming might be a dysfunctional coping strategy to alleviate negative feelings and stressful situations [12, 13].

### Emotion regulation

The concept of emotion regulation (ER) and its association with psychopathology have been intensively studied during the last years [14–16]. Tull and Aldao differentiate between ER abilities and strategies [17]. The ability to recognize, understand and regulate one’s own emotions is seen as dispositional and describes the typical way in which people experience their emotions. Therefore, it focuses primarily on the person’s general regulation potential while ER strategies like reappraisal, suppression or procrastination refer to specific behaviors that actively influence the experience or expression of emotions and can be directly targeted in psychotherapy [18]. The maladaptive strategy procrastination describes the delay of necessary or important activities even though the postponement of these obligations results in negative consequences [19] and is seen as a failure of self-regulatory competencies [20]. Current research indicates that the general procrastination tendency of a person is a relatively stable trait, even though contextual factors might influence the degree of procrastination [21]. Accordingly, difficulties in ER are associated with mental disorders including behavioral addictions [22, 23].

The development of ER continues into early adulthood [24, 25]. Contrary to a linear assumption that the efficacy of ER skills grows with age, current research suggests that there is a major reorganization of ER during adolescence with an increased use of maladaptive strategies [25–27]. These findings emphasize the severe challenges adolescents face during puberty—a critical developmental period with a high vulnerability for mental disorders in general [24]. Moreover, neurobiological evidence indicate that immature prefrontal and limbic regions promote insufficient emotion regulation and might therefore be especially affected by conflicting emotions in decision-making processes [24].

### Problematic gaming and emotion regulation

Cross-sectional studies could find an association between emotional dysregulation and PG among adolescents [23, 28–31]. Regarding the maladaptive ER strategy procrastination, there are hints for a positive association between high levels of procrastination and the clinical severity of PG in young adults [32]. First longitudinal studies highlight that ER difficulties could predict PG [33–35].

However, the listed studies display various limitations: firstly, comparability of studies and generalizability is limited due to varying definitions of PG not including all diagnostic criteria of DSM-5 or ICD-11 [23, 28, 31–33] and only one study having investigated a representative sample [31]. Moreover, the assessment of ER does not implement the differentiation in ER abilities and strategies proposed by Tull and Amendola (2015) and, therefore, does not capture the concept in its full complexity [30, 31, 34]. Among children and adolescents the current research supports an association between procrastination and problematic social media or internet use [36–38]. To the best of our knowledge, procrastination and its specific association with PG in adolescents have not yet been examined. Furthermore, no differentiated analyses on ER and PG accounted for adolescent age groups (older children vs. youths) and gender as well as the time course of gaming patterns.

### The present study

From a developmental perspective, it remains an open question which ER aspects specifically promote or maintain the occurrence of PG and its manifestations as hazardous or disordered gaming in adolescence. The current study aimed to close a significant gap in the understanding of ER difficulties and PG in a critical age group. For the first time, a representative sample of older children (10–13 years) and youths (14–17 years) was investigated regarding symptoms of PG based on standardized DSM-5/ICD-11 criteria and ER abilities from a cross-sectional and prospective perspective. Different ER strategies, the effects of age groups and gender as well as the development of different gaming patterns over time were considered to identify specific risk factors for a better understanding of PG and detect potential targets in individualized prevention and treatment measures.

## Materials and methods

### Participants and procedures

The current study was part of a large representative online survey on digital media use among adolescents and conducted with the help of the established German market and opinion polling company forsa in September 2019 and November 2020. Initially, 23,716 adults between the ages of 28 and 75 were contacted via e-mail with a response-rate of 12,427 individuals. 1733 of these households reported to have children between the ages of 10 and 17. After asking them to participate, 1221 adolescents agreed to be part of the study and completed the questionnaires at the first measurement point. 659 of those participated in the follow-up. In terms of age, gender and region of residence, representativity of the proportion was given. Two adolescent age groups were considered based on the German social code (“Sozialgesetzbuch”) defining children as being younger than 14 years and youths as being younger than 18 years [39]. All adolescents and their caregivers provided informed consent prior to the participation and could withdraw from the study at any time. Participants did not receive any compensation. The overall average response time to complete all questionnaires was 26 min including breaks. Both national and international ethical guidelines, in accordance with the Declaration of Helsinki, were followed in the realization of the study. The “Local Psychological Ethics Commission at the Center for Psychosocial Medicine” (LPEK) of the “University Medical Center Hamburg Eppendorf” (UKE) gave its approval.

### Measures

#### Problematic gaming

The Internet Gaming Disorder Scale (IGDS) by Lemmens et al. [40], based on the DSM-5 criteria for IGD [40], was used to assess PG symptoms among adolescents in the baseline-sample. Composed of nine questions with binary answer options (0 = “no”/1 = “yes”), the cut-off for pathological gaming was reached at five or more points. Accordingly, higher scores in the IGDS indicated more severe PG. The questionnaire was repeatedly used among German adolescents and showed an overall suitability and validity to identify IGD among this age group on a population level [39]. In the baseline sample, Cronbach’s α for the IGDS was 0.85, indicating a good internal consistency.

The Gaming Disorder Scale for Adolescents (GADIS-A), an instrument created by Paschke et al. [6], was used to assess PG based on the ICD-11 criteria of GD and HG [6]. It comprises two factors, cognitive behavioral symptoms and negative consequences, combined with a time criterion. The questionnaire was composed of nine statements regarding the symptomatology with response options on a five-point Likert-scale (0 = “strongly disagree” to 4 = “strongly agree“). An additional item, the time criterion, assessed the frequency of symptoms with four response options (0 = “not at all” to 3 = “almost daily”). GD was assumed, if the cut-offs for both factors were reached and the time criterion was fulfilled. However, if the time criterion and/or the cut-off value for negative consequences were not reached, HG was indicated [6]. Cronbach’s α values of 0.93 in the follow-up sample (factor 1 “cognitive behavioral symptoms” = 0.89; factor 2 “negative consequences” = 0.90) demonstrated an excellent internal consistency.

The development of gaming patterns between the two measurement points was described by four categories: (1) no or unproblematic gaming behavior (IGDS < cut-off at baseline and GADIS-A < cut-offs at follow-up); (2) remission of PG (IGDS ≥ cut-off at baseline and GADIS-A < cut-offs at follow-up); (3) constant PG (IGDS ≥ cut-off at baseline and GADIS-A ≥ cut-offs at follow-up); (4) new PG (IGDS < cut-off at baseline and GADIS-A ≥ cut-offs at follow-up).

### Emotional dysregulation

Emotional dysregulation was assessed through the short form of the Difficulties in Emotional Regulation Scale (DERS-SF) by Kaufman et al. [41]. In this widely used 18-item measure with response options on a five-point Likert-scale (1 = “almost never” to 5 = “almost always”), higher scores indicated greater emotional regulation difficulties. In the past, the instrument has demonstrated a good fit for adolescents [42, 43]. The internal consistency for the total questionnaire among the baseline-sample was good (Cronbach’s *α* = 0.90). Six subscales were differentiated with excellent to questionable internal consistency in the present sample: deficits in emotional awareness (Cronbach’s *α* = 0.67), lack of emotional clarity (Cronbach’s *α* = 0.81), non-acceptance of emotional responses (Cronbach’s *α* = 0.71), deficits in engaging in goal-directed behavior (Cronbach’s *α* = 0.84), difficulties in impulse control (Cronbach’s *α* = 0.90) and limited access to emotion regulation strategies (Cronbach’s *α* = 0.78) [14]. Due to the novel differentiated ER strategy approach with respect to PG, the subscale emotional awareness was kept for further analysis although it’s internal consistency was below the threshold that is regarded as acceptable (Cronbach’s *α* > 0.70) [44].

The Procrastination Questionnaire for Students (PFS-4) [45] was used to measure tendencies of behavioral avoidance, a short-term (maladaptive) emotion regulation strategy. Higher values in the PFS-4 indicated stronger tendencies to procrastinate [46]. Initially, it was validated among German university students [45] but due to its simple structure with four items, answered on a five-point Likert-scale (1 = “[almost] never” to 5 = “[almost] always”) related to academic tasks, it could prove suitability for high school students in clinical and research settings [47]. Moreover, an excellent internal consistency in the baseline sample further supported the use among adolescents (Cronbach’s *α* = 0.90).

### Data analyses

All statistical analyses were performed with the software package R version 4.0.3 [48]. The data was analyzed calculating absolute and relative frequencies with 95% confidence intervals for categorical variables and mean values with standard deviations for metric variables with the statistical package *psych*. To account for the right-skewed distribution of IGDS and GADIS-A scores, Poisson regression models were computed for the cross-sectional and the longitudinal analyses (package *stats*). Adolescent age groups (children vs. youths) and gender were included as covariates. DERS and PFS scores were z-scaled for easier interpretability. Moreover, general logistic regression models were estimated to differentiate between the different patterns of PG over time. Finally, a multinomial logistic regression analyzed predictors for different gaming patterns (no gaming, HG and GD compared to frequent, but unproblematic gaming behavior; R package *nnet*). All model requirements have been carefully reviewed before analysis.

## Results

### Sample characteristics

Demographic, emotion-regulation, and gaming pattern characteristics for the baseline and follow-up survey are presented in Table 1.

**Table 1:** Sample characteristics

Categories	Baseline sample	Follow-up sample
	*N* (%)/mean (± SD; range)	
Sociodemographic measures		
Absolute frequency	1221	659
Children (10–13 years)	720 (58.97%)	378 (57.36%)
Youths (14–17 years)	501 (41.03%)	281 (42.64%)
Age in years	13.04 (2.39; 10–17)	13.11 (2.38; 10–17)
Gender		
Female	563 (46.11%)	308 (46.74%)
Male	658 (53.89%)	351 (53.26%)
Place of residence		
Rural living	249 (20.39%)	111 (16.84%)
Urban living	972 (79.61%)	548 (83.16%)
School attendance		
Yes	1132 (92.71%)	601 (91.2%)
No	88 (7.21%)	58 (8.80%)
Educational level (EL)		
Low	99 (8.11%)	54 (8.19%)
Middle or high	1069 (87.55%)	579 (87.86%)
Gaming pattern		
IGDS score (sum)	1.03 (2.46; 0 – 9)	1.87 (2.49; 0–9)
IGDS-based gaming groups at baseline		
Unproblematic gaming (< 5)	1022 (83.7%)	545 (83.72%)
Problematic gaming (≥ 5)	199 (16.3%)	106 (16.28%)
GADIS-A score (sum) at follow-up	6.61 (6.74; 0–36)	5.93 (6.81; 0–36)
Cognitive behavioral symptoms (sum)		2.05 (3.36; 0–20)
Negative consequences (sum)		3.88 (3.98; 0–20)
GADIS-A-based gaming groups at follow-up		
No frequent gaming		98 (14.87%)
Unproblematic gaming		477 (72.38%)
Hazardous gaming (HG)		69 (10.47%)
Gaming disorder (GD)		8 (1.21%)
Emotion regulation measures		
DERS score (sum)	40.47 (12.46; 18–82)	39.55 (12.28; 18–82)
DERS subscales		
Strategies	6.30 (2.83; 3–15)	6.19 (2.87; 3–15)
Non-acceptance	6.30 (2.83; 3–15)	6.25 (2.79; 3–15)
Impulsiveness	5.78 (3.06; 3–15)	5.63 (3.04; 3–15)
Goal-oriented behavior	7.21 (3.08; 3–15)	6.98 (3.00; 3–15)
Clarity	6.48 (2.82; 3–15)	6.40 (2.80; 3–15)
Awareness	8.39 (2.89; 3–15)	8.27 (2.85; 3–15)
PFS score (sum)	11.08 (4.03; 4–20)	10.91 (4.00; 4–20)

Missing values: migration background t0—*n* = 25 (0.9%), t1—*n* = 1 (0.15%), EL t0—*n* = 53 (4.34%), t1—*n* = 26 (3.95%), gaming group t0—*n* = 14 (1.15%), gaming group t1—*n* = 7 (1.06%)

*SD* standard deviation, *EL* estimated educational degree of the participant; *EL low* no, special school (“Förderschulabschluss”) or lower school certificate (“Hauptschulabschluss”), *EL middle/high* secondary school certificate (“Realschulabschluss”) to university entry qualification (“Abitur”); equivalence testing revealed no differences in the sample characteristics between baseline- and follow-up sample, *IGDS* internet gaming disorder scale, *GADIS-A* gaming disorder scale for adolescents, *DERS* difficulties in emotion regulation scale, *PFS* procrastination questionnaire for students

### Cross-sectional analyses

#### General Poisson model

To evaluate the influence of emotional dysregulation on PG while controlling for age groups and gender, we conducted a multivariate general Poisson regression analysis (see Table 2). Both ER measures, based on DERS and PFS score, and the covariates were significantly associated with more symptoms of PG. The overall model showed a variance explanation of 59.9% (*R*^2^ Nagelkerke = 0.60).

**Table 2:** Emotion regulation characteristics as risk factors for problematic gaming in adolescents

IGDS score			
Predictors	Incidence rate ratios	CI	*p*
(Intercept)	0.14	0.11–0.19	< 0.001***
DERS sum score	1.36	1.27–1.46	< 0.001***
PFS sum score	1.34	1.24–1.44	< 0.001***
Covariates			
Gender (boys)	2.00	1.73–2.30	< 0.001***
Age group (children)	1.39	1.21–1.59	< 0.001***
Observations	1128		
*R*^2^ Nagelkerke	0.599		

*IGDS* internet gaming disorder scale, *DERS* difficulties in emotion regulation scale, *PFS* procrastination questionnaire for students, DERS and PFS sum scores are z scaled, *CI* Confidence interval; age groups were comprised of children (10–13 years) and youths (14–17 years), level of significance: p* < 0.05, p** < 0.01, p*** < 0.001

#### Subsample analyses at baseline

To gain further insight into the developmental role of ER on PG, different ER aspects were separately investigated for age groups (while controlling for gender; Table 3). For children, a lack of emotional clarity and deficits in emotional awareness were significantly associated with more PG scores. For youths, however, higher values in non-acceptance of emotional responses were a significant predictor for PG symptoms. Higher procrastination and male gender (covariate) were significantly associated with PG in both subsamples.

**Table 3:** Differential emotion regulation characteristics as risk factors for problematic gaming: children vs. youths

IGDS score							
Children				Youths			
Predictors	Incidence rate ratios	CI	*p*	Predictors	Incidence rate ratios	CI	*p*
(Intercept)	0.19	0.13–0.28	< 0.001***	(Intercept)	0.12	0.07–0.20	< 0.001***
DERS subscales				DERS subscales			
Strategies	1.14	1.00–1.29	0.058	Strategies	1.04	0.87–1.24	0.648
Nonacceptance	0.98	0.88–1.10	0.726	** Nonacceptance**	1.20	1.02–1.41	0.027*
Impulsivity	0.99	0.89–1.10	0.849	Impulsivity	1.04	0.90–1.20	0.629
Goal-oriented behavior	1.13	0.99–1.28	0.064	Goal-oriented behavior	1.15	0.97–1.36	0.116
** Clarity**	1.17	1.05–1.29	0.003**	Clarity	1.02	0.88–1.18	0.795
** Awareness**	1.12	1.03–1.22	0.010**	Awareness	1.02	0.90–1.15	0.792
**PFS**	1.32	1.20–1.44	< 0.001***	**PFS**	1.31	1.15–1.50	< 0.001***
Covariate				Covariate			
**Gender (boys)**	1.69	1.42–2.01	< 0.001***	**Gender (boys)**	2.99	2.25–3.98	< 0.001***
Observations	627			Observations	462		
*R*^2^ Nagelkerke	0.568			*R*^2^ Nagelkerke	0.640		

Age groups were comprised of children (10–13 years) and youths (14–17 years), level of significance: p*< 0.05, p** < 0.01, p*** <0.001

*IGDS* internet gaming disorder scale, *DERS* difficulties in emotion regulation scale, *PFS* procrastination questionnaire for students, DERS and PFS sum scores are z scaled, *CI* Confidence interval

With regard to gender differences (controlled for age group), a subgroup analysis between girls and boys showed that more deficits in goal-directed behavior and problems with emotional awareness in boys was associated with higher PG scores. For girls on the other hand, significant predictors for higher PG scores were greater non-acceptance of emotional responses and a lack of emotional clarity. Higher scores for procrastination were significantly associated with higher PG scores among both genders (see Table 4).

**Table 4:** Differential emotion regulation characteristics as risk factors for problematic gaming: boys vs. girls

IGDS score							
Boys				Girls			
Predictors	Incidence rate ratios	CI	*p*	Predictors	Incidence Rate Ratios	CI	*p*
(Intercept)	0.32	0.22–0.48	< 0.001***	(Intercept)	0.09	0.05–0.18	< 0.001***
DERS subscales				DERS subscales			
Strategies	1.06	0.90–1.20	0.311	Strategies	1.14	0.93–1.41	0.213
Nonacceptance	1.00	0.90–1.11	0.976	**Nonacceptance**	1.23	1.03–1.47	0.025*
Impulsivity	1.04	0.94–1.16	0.395	Impulsivity	0.97	0.83–1.14	0.751
** Goal-oriented behavior**	1.15	1.02–1.29	0.019*	Goal-oriented behavior	1.07	0.89–1.30	0.467
Clarity	1.07	0.97–1.18	0.172	**Clarity**	1.17	1.01–1.37	0.043*
** Awareness**	1.13	1.05–1.23	0.002**	Awareness	0.95	0.82–1.10	0.488
**PFS**	1.31	1.20–1.44	< 0.001***	**PFS**	1.30	1.14–1.50	< 0.001***
Covariate				Covariate			
Age group (children)	1.15	0.97–1.35	0.100	**Age group (children)**	2.06	1.54–2.77	< 0.001***
Observations	581			Observations	508		
*R*^2^ Nagelkerke	0.492			*R*^2^ Nagelkerke	0.528		

Age groups children comprised of participants from 10 to 13 years, level of significance: p* < 0.05, p** < 0.01, p *** < 0.001

*IGDS* internet gaming disorder scale, *DERS* difficulties in emotion regulation scale, *PFS* procrastination questionnaire for students, DERS and PFS sum scores are z scaled, *CI* Confidence interval

### Longitudinal analyses

#### Longitudinal general Poisson model

Risk factors for prospective PG were identified estimating a general Poisson model in the 14-month-follow-up sample. Accordingly, the influence of emotional dysregulation on PG based on the ICD-11 criteria of GD were investigated while controlling for the gaming pattern at baseline, gender, and age group. Higher GADIS-A-scores after one year were significantly predicted by higher scores on both emotional dysregulation scales at baseline (see Table 5). Moreover, baseline IGDS scores served as a significant covariate whereas the variables age group and gender did not. The overall model explained a total variance of 87.7% (*R*^2^ Nagelkerke = 0.88).

**Table 5:** General Poisson model: Risk factors for problematic gaming among adolescents after one year

GADIS-A total score			
Predictors	Incidence rate ratio	CI	*p*
(Intercept)	1.44	1.02–2.04	0.037*
**DERS (sum)**	1.17	1.07–1.28	0.001**
**PFS (sum)**	1.16	1.06–1.27	0.002**
Covariates			
**IGDS (sum)**	1.37	1.27–1.49	< 0.001***
Gender (male)	1.17	0.99–1.38	0.066
Age group (child)	1.04	0.88–1.23	0.628
Observations	607		
*R*^2^ Nagelkerke	0.877		

*GADIS-A* gaming disorder scale for adolescents, *DERS* difficulties in emotion regulation scale, *PFS* procrastination questionnaire for students, *IGDS* internet gaming disorder scale, *CI* Confidence interval, DERS and PFS sum scores are z scaled, level of significance: p* < 0.05, p** <0.01, p*** <0.001

#### Emotion regulation and prospective stability of problematic gaming

Based on the follow-up investigation after 14 months, four different gaming groups were identified. Their sample characteristics are presented in Table 6.

**Table 6:** Characteristics of adolescents with different gaming patterns over time

Categories	No PG	Remission	Constant PG	New PG
	*N* (%)/mean (± SD; range)			
N^a^	499 (75.72%)	73 (11.10%)	31 (4.70%)	38 (5.77%)
Age	13.24 (2.42; 10–17)	12.52 (2.14; 10–17)	13.0 (2.35; 10–17)	12.74 (2.04; 10–17)
Gender				
Female	257 (51.5%)	21 (28.77%)	8 (25.81%)	14 (36.84%)
Male	242 (48.5%)	52 (71.23%)	23 (74.19%)	24 (63.16%)
Region				
Rural	83 (16.63%)	13 (17.81%)	6 (19.35%)	7 (18.42%)
Urban	416 (83.37%)	60 (82.19%)	25 (80.65%)	31 (81.58%)
Education				
Low	34 (7.04%)	11 (15.49%)	2 (6.9%)	5 (13.51%)
Middle/High	449 (92.96%)	60 (84.51%)	27(93.1%)	32 (86.49%)

*PG:* problematic gaming, *No PG:* No PG at t0 and t1, *Remission:* PG at t0, no PG at t1, *Constant PG:* PG at t0 and t1, *New PG:* No PG at t0, PG at t1, *SD* standard deviation, *EL* estimated educational degree of participants, *Education low* no, special school (“Förderschulabschluss”) or lower school certificate (“Hauptschulabschluss”), *Education middle/high* secondary school certificate (“Realschulabschluss”) to university entry qualification (“Abitur”)

^a^No classification due to severe missing values *n* = 18 (2.73%)

By estimating a logistic regression model, the group of adolescents with new PG was compared to participants without PG (see Table 7). Age and gender could not be identified as significant covariates in the general longitudinal Poisson model (after controlling for baseline gaming patterns) and were therefore not considered in the logistic regression model. The DERS total score reached the level of significance when comparing new PG to no PG groups. Accordingly, higher scores in the DERS increased the odds of developing new PG behavior in the follow-up investigation by 1.83. Moreover, the group of remitted gamers were compared to participants with constant PG over the two measurement points to identify variables maintaining PG. In this model, lower procrastination scores increased the probability of being categorized into the group of remitted gamers significantly among the follow-up sample (see Table 8).

**Table 7:** Logistic regression model: emotion regulation characteristics as risk factors for new problematic gaming in adolescents after 14 months

I. New PG vs. No PG			
Predictors	Odds ratio	CI	*p*
(Intercept)	0.01	0.00–0.02	< 0.001***
**DERS (sum)**	1.83	1.29–2.57	0.001**
PFS (sum)	1.13	0.79–1.63	0.494
Observations	511		
*R*^2^ Nagelkerke	0.036		

*PG* problematic gaming, *No PG* No PG at t0 and t1, *New PG* No PG at t0, PG at t1, *CI* confidence interval, *DERS* difficulties in emotion regulation scale, *PFS* procrastination questionnaire for students, DERS and PFS are z-scaled, level of significance: p* < 0.05, p** < 0.01, p*** < 0.001

**Table 8:** Logistic regression model: emotion regulation characteristics to predict remission of problematic gaming in adolescents after 14 months

II. Remission vs. constant PG			
Predictors	Odds ratio	CI	*p*
(Intercept)	11.79	1.25–111.46	0.031*
DERS (sum)	1.06	0.66–1.70	0.823
**PFS (sum)**	0.60	0.36–0.98	0.043*
Observations	93		
*R*^2^ Nagelkerke	0.051		

*PG* problematic gaming, *Remission* PG at t0, no PG at t1, *Constant PG* PG at t0 and t1, *CI* confidence interval, *DERS* difficulties in emotion regulation scale, *PFS* procrastination questionnaire for students, DERS and PFS are z-scaled, level of significance: p* < 0.05, p** < 0.01, p*** < 0.001

#### Emotion regulation and gaming patterns at follow-up

Finally, a multinomial logistic regression was conducted to examine the differences between gaming patterns according to ICD-11 definitions (see Table 9). While among hazardous gamers, both ER measurements were significant, for participants with a manifest GD only the DERS total score was a significant predictor.

**Table 9:** Multinomial logistic regression: emotion regulation characteristics and prospective gaming group

Gaming groups (compared to unproblematic gaming)			
Predictors	Odds ratio	CI	*p*
*No gaming*			
(Intercept)	0.13	0.06–0.32	< 0.001***
DERS (sum)	1.25	0.97–1.63	0.087
PFS (sum)	0.88	0.67–1.14	0.330
*Hazardous gaming (HG)*			
(Intercept)	0.01	0.00–0.03	< 0.001***
** DERS (sum)**	1.60	1.18–2.16	0.003**
** PFS (sum)**	1.43	1.05–1.96	0.025*
*Gaming disorder (GD)*			
(Intercept)	0.00	0.00–0.00	< 0.001***
** DERS (sum)**	4.46	1.80–11.02	0.001**
PFS (sum)	1.81	0.73–4.49	0.199
Observations	604		
*R*^2^ Nagelkerke	0.220		

*CI* confidence interval, *DERS* difficulties in emotion regulation scale, *PFS* procrastination questionnaire for students, DERS and PFS were z-scaled, level of significance: p* < 0.05, p** < 0.01, p*** < 0.001

## Discussion

Within the present study, emotional dysregulation as a potential risk factor for PG was investigated in detail in a representative sample of adolescents while accounting for the PG criteria of the two most influential classifications systems as well as for age and gender effects from a cross-sectional and prospective perspective for the first time. Accordingly, risk factors for children and adolescents as well as for boys and girls with regard to their ER competencies could be identified. By implementing the ICD-11 criteria it was possible to distinguish ER factors contributing to HG or GD separately [24]. Lee and colleagues (2017) claim that PG should be seen as a heterogenous disorder and identify different subtypes [13]. Therefore, besides an impulsive-aggressive and a socially conditioned type, they discuss a subgroup with emotionally vulnerable traits using gaming as an escape or coping strategy [13]. Hence, emotional distress might trigger those adolescents, lacking efficient ER competencies and then result in excessive gaming. Additionally, the I-PACE-model, developed by Brand and colleagues (2016) emphasizes the relevance of deficient ER processes in gaming based on neurobiological evidence indicating an imbalance between ER circuits and cognitive flexibility [49]. Furthermore, alexithymia, the inability to describe and name emotions both in oneself and others, is found to be associated with PG among young adults [50]. Consistent with these findings, the present data suggested that difficulties in cognitive and behavioral ER processes, including greater tendencies to procrastinate, represented risk factors for the development of PG in children and adolescents cross-sectionally as well as prospectively. With the combination of the DERS-SF and PFS-4 it was possible to depict a broad picture of the different dimensions underlying emotional dysregulation based on the concept of ER abilities and strategies [17, 18].

ER characteristics as risk factors for more PG symptoms differed between children and adolescents. While for children difficulties in clearly identifying and being aware of their own emotions seemed to be most relevant, for adolescents it was their acceptance. Emotional clarity and awareness are found to be foundational for every further aspect of ER [51] and therefore pose a relevant developmental task for children. Previous research indicates that awareness of one’s emotions is a metacognitive task that children are not yet capable of [52], which is partly explained by premature executive control functions [22, 52, 53]. Moreover, the children’s age group was a significant covariate for more PG symptoms in the baseline indicating the importance to consider potential age effects. This link might be explained by neurostructural and neurofunctional similarities of immature ER and PG, especially among prefrontal and frontolimbic regions [10, 24]. Given limited available research findings on age and PG [9], further studies should look at adolescent age groups more closely.

Additionally, the present study might add important aspects to the repeatedly replicated gender differences on PG prevalence with boys being affected more often [9, 54]. A decreased awareness of emotions was shown to be a significant risk factor for more PG symptoms in boys, as known from previous research on gender differences among ER processes [55, 56]. Developmental research indicates that the beginning of puberty begins in boys about 2 years later than in girls [57]. Deficits among boys might be partly favored by a delayed onset of puberty and therefore immature executive control functions. If girls’ cognitive capacities developed earlier than boys’, higher difficulties in accepting emotions rather than recognizing them by girls, and similar to the youths age group, could be explained. According to a bio-psycho-social framework, gender differences in ER processes emerge through a combination of biological differences, social learning theories and the specific interactions of social contexts and expectations [58]. Therefore, in line with previous findings [59], the ER strategies and abilities substantially differed between gender. Interestingly, a significant association between procrastination and PG in adolescents was described for the first time. This maladaptive ER strategy seems to be an important risk factor for PG among children and youths as well as in boys and girls that should be specifically addressed in therapy.

Due to the longitudinal approach, it was possible to observe the development of gaming behavior over time. On the one hand, results of a logistic regression indicated that difficulties in ER promoted the emergence of PG. On the other hand, data from adolescents with remitted gaming compared to constant problematic gaming behavior identified the maladaptive ER strategy procrastination to be a maintaining factor for PG. The approach of a more detailed examination of gaming behavior over time with the stratification into different gaming groups has been rarely applied in the currently available research. One longitudinal study analyzed four different gaming patterns among adolescents based on DSM-IV addiction criteria [33]. The authors postulate that the emergence of PG in adolescents is associated with higher impulsivity, lower social competence and empathy, and poorer ER skills [33]. Moreover, the comparison of different gaming groups over time is implemented in a study by Tsai et al. (2020) among young college students with an internet addiction [60]. The authors suggest that higher impulsivity promotes the development of new addictive behavior [60]. Future research should further investigate the development of PG behavior over time to specifically identify facilitating and maintaining factors and gain insight into the temporal stability of PG based on remission rates.

Finally, another strength of this study was the differentiation between normal and hazardous gaming behavior as well as a manifest gaming disorder according to the ICD-11 criteria. Interestingly, difficulties in the ER strategy procrastination seemed to affect especially adolescents who are at risk of developing PG. However, emotional dysregulation in terms of difficulties with the ER abilities influenced adolescents with hazardous gaming behavior as well as a manifest gaming disorder. Yet, it must be noted, that the sample size of adolescents with GD according to the GADIS-A in the follow-up sample was very small (*n* = 8). Nevertheless, a strong effect could be seen which underlines the importance of impaired ER abilities in PG.

By combining DSM-5 and ICD-11 approaches, a broad screening as well as a specific look of different gaming patterns was achieved and a high correlation between both instruments over time could be shown. With the ICD-11 definition, the impairment of the behavior was crucial and, therefore, especially important for the clinical relevance of the symptomatology among adolescents and the presented findings.

### Limitations

Although current research indicate that depression, anxiety or ADHD are closely linked to PG [61], survey participants’ comorbidities could not be considered. Accordingly, accompanied mental disorders might have confounded the ability to regulate emotions [62]. In this respect, even though procrastination is not a diagnostic criterion of ADHD, a study among young adults indicates an association between ADHD and greater procrastination scores. Therefore, an assessment of these comorbidities would have been even more relevant [63]. While securing a representative sample was a goal in terms of age, gender and region of residence, the use of online-surveys required internet access which cannot be guaranteed in approximately 5% of the German households [64]. Additionally, a true representativity is uncertain due to unknown factors that might determine who is willing to take online-surveys in the first place. Moreover, households with insufficient knowledge of German could have been neglected because the language in the administered questionnaires was German. Even though equivalence testing of the sample characteristics revealed no significant differences between baseline- and follow-up sample, there were approximately 50% less participants in the follow-up investigation which might have influenced the results as well. A common methodological problem is the use of self-reports due to errors in recollection or socially desired answers. Even though participants were asked to complete the questionnaires on their own, influences from third parties cannot be ruled out completely. Therefore, future studies should consider additional parental questionnaires to complement the assessment [e.g., GADIS-P, [65]] and clinical interviews as the gold standard for a PG diagnosis. Moreover, internal consistency of all standardized scales was assessed using Cronbach’s α. The DERS subscale emotional awareness could not reach a sufficient value. Yet, since internal consistency is necessary, but not sufficient for validity and Cronbach’s α reflects not only scale property but also sample attributes [66], we decided to leave this subscale in the analyses. Given the early state of research on ER and PG, this is reasonable but should be kept in mind during interpretation of the results.

### Clinical implications

Difficulties with ER in general were found to be predictors of PG. Therefore, the present findings support the inclusion of specific ER trainings in prevention and intervention of PG [67, 68]. However, given the present findings on the role for different ER aspects in boys and girls as well as in children and youths, a tailored approach is warranted including mindfulness-based cognitive therapy, dialective behavioral therapy, or acceptance-based behavioral therapy [69].

## Conclusion

Emotional dysregulation in general and procrastination as one specific ER strategy could be shown to be strong predictors for PG across adolescent age groups and gender. With regard to problematic emotion regulation strategies, gender and age differences are evident. While children have difficulty recognizing emotions, adolescents have more problems accepting them. Interestingly, boys seem to have difficulties in the awareness of their emotions, while girls, that are usually further along with their cortical development, show more problems with the acceptance of their own emotions. Moreover, emotional dysregulation including procrastination could predict different gaming patterns and their stability after 14 months.

## Data Availability

After all results of the parent–child survey will be published, the data can be provided upon request by the corresponding author (K.P.).

## References

[1] King DL, Delfabbro PH, Billieux J, Potenza MN (2020). Problematic online gaming and the COVID-19 pandemic. J Behav Addict.

[2] Paschke K, Austermann MI, Simon-Kutscher K, Thomasius R (2021). Adolescent gaming and social media usage before and during the COVID-19 pandemic. SUCHT.

[3] Stevens MW, Dorstyn D, Delfabbro PH, King DL (2021). Global prevalence of gaming disorder: a systematic review and meta-analysis. Aust N Z J Psychiatry.

[4] American Psychiatric Association (2013). Diagnostic and statistical manual of mental disorders (DSM-5).

[5] World Health Organization (2017) International Classification of Diseases 11th Revision ICD-11

[6] Paschke K, Austermann MI, Thomasius R (2020). Assessing ICD-11 gaming disorder in adolescent gamers: development and validation of the gaming disorder scale for adolescents (GADIS-A). J Clin Med.

[7] Jo YS, Bhang SY, Choi JS (2019). Clinical characteristics of diagnosis for internet gaming disorder: comparison of DSM-5 IGD and ICD-11 GD diagnosis. J Clin Med.

[8] Sugaya N, Shirasaka T, Takahashi K, Kanda H (2019). Bio-psychosocial factors of children and adolescents with internet gaming disorder: a systematic review. Biopsychosoc Med.

[9] Paulus FW, Ohmann S, Gontard A, Popow C (2018). Internet gaming disorder in children and adolescents: a systematic review. Dev Med Child Neurol.

[10] Schettler L, Thomasius R, Paschke K (2021). Neural correlates of problematic gaming in adolescents: a systematic review of structural and functional magnetic resonance imaging studies. Addict Biol.

[11] Nielsen P, Favez N, Rigter H (2020). Parental and family factors associated with problematic gaming and problematic internet use in adolescents: a systematic literature review. Curr Addict Rep.

[12] Kardefelt-Winther D (2014). A conceptual and methodological critique of internet addiction research: towards a model of compensatory internet use. Comput Hum Behav.

[13] Lee S-Y, Lee HK, Choo H (2017). Typology of Internet gaming disorder and its clinical implications. Psychiatry Clin Neurosci.

[14] Gratz KL, Roemer L (2004). Multidimensional assessment of emotion regulation and dysregulation: development, factor structure, and initial validation of the difficulties in emotion regulation scale. J Psychopathol Behav Assess.

[15] Thompson RA (2019). Emotion dysregulation: a theme in search of definition. Dev Psychopathol.

[16] Gross JJ (2015). The extended process model of emotion regulation: elaborations, applications, and future directions. Psychol Inq.

[17] Tull MT, Aldao A (2015). Editorial overview: new directions in the science of emotion regulation. Curr Opin Psychol.

[18] Weiss NH, Kiefer R, Goncharenko S (2022). Emotion regulation and substance use: a meta-analysis. Drug Alcohol Depend.

[19] Klingsieck KB (2013). Procrastination. Eur Psychol.

[20] Rebetez MML, Rochat L, Barsics C, van der Linden M (2018). Procrastination as a self-regulation failure: the role of impulsivity and intrusive thoughts. Psychol Rep.

[21] Johansson F, Rozental A, Edlund K (2023). Associations between procrastination and subsequent health outcomes among university students in Sweden. JAMA Netw Open.

[22] Aldao A, Nolen-Hoeksema S, Schweizer S (2010). Emotion-regulation strategies across psychopathology: a meta-analytic review. Clin Psychol Rev.

[23] Estévez A, Jáuregui P, Sánchez-Marcos I (2017). Attachment and emotion regulation in substance addictions and behavioral addictions. J Behav Addict.

[24] Ahmed SP, Bittencourt-Hewitt A, Sebastian CL (2015). Neurocognitive bases of emotion regulation development in adolescence. Dev Cogn Neurosci.

[25] Zimmermann P, Iwanski A (2014). Emotion regulation from early adolescence to emerging adulthood and middle adulthood. Int J Behav Dev.

[26] Cracco E, Goossens L, Braet C (2017). Emotion regulation across childhood and adolescence: evidence for a maladaptive shift in adolescence. Eur Child Adolesc Psychiatry.

[27] Lange S, Tröster H (2015). Adaptive und maladaptive Emotionsregulationsstrategien im Jugendalter. Zeitschrift für Gesundheitspsychologie.

[28] Uçur Ö, Dönmez YE (2020). Problematic internet gaming in adolescents, and its relationship with emotional regulation and perceived social support. Psychiatry Res.

[29] Amendola S, Spensieri V, Guidetti V, Cerutti R (2019). The relationship between difficulties in emotion regulation and dysfunctional technology use among adolescents. J Psychopathol.

[30] Schneider LA, King DL, Delfabbro PH (2018). Maladaptive coping styles in adolescents with internet gaming disorder symptoms. Int J Ment Health Addict.

[31] Kökönyei G, Kocsel N, Király O (2019). The role of cognitive emotion regulation strategies in problem gaming among adolescents: a nationally representative survey study. Front Psych.

[32] Yeh Y-C, Wang P-W, Huang M-F (2017). The procrastination of Internet gaming disorder in young adults: the clinical severity. Psychiatry Res.

[33] Gentile DA, Choo H, Liau A (2011). Pathological video game use among youths: a two-year longitudinal study. Pediatrics.

[34] Liau AK, Choo H, Li D (2015). Pathological video-gaming among youth: A prospective study examining dynamic protective factors. Addict Res Theory.

[35] Wichstrøm L, Stenseng F, Belsky J (2019). Symptoms of internet gaming disorder in youth: predictors and comorbidity. J Abnorm Child Psychol.

[36] Wartberg L, Thomasius R, Paschke K (2021). The relevance of emotion regulation, procrastination, and perceived stress for problematic social media use in a representative sample of children and adolescents. Comput Hum Behav.

[37] Reinecke L, Meier A, Beutel ME (2018). The relationship between trait procrastination, internet use, and psychological functioning: results from a community sample of German adolescents. Front Psychol.

[38] Traş Z, Gökçen G (2020). Academic procrastination and social anxiety as predictive variables internet addiction of adolescents. IES.

[39] Paschke K, Sack P-M, Thomasius R (2021). Validity and psychometric properties of the internet gaming disorder scale in three large independent samples of children and adolescents. Int J Environ Res Public Health.

[40] Lemmens JS, Valkenburg PM, Gentile DA (2015). The internet gaming disorder scale. Psychol Assess.

[41] Kaufman EA, Xia M, Fosco G (2016). The difficulties in emotion regulation scale short form (DERS-SF): validation and replication in adolescent and adult samples. J Psychopathol Behav Assess.

[42] Weinberg A, Klonsky ED (2009). Measurement of emotion dysregulation in adolescents. Psychol Assess.

[43] Neumann A, van Lier PAC, Gratz KL, Koot HM (2010). Multidimensional assessment of emotion regulation difficulties in adolescents using the difficulties in emotion regulation scale. Assessment.

[44] Nunnally JC, Bernstein IH (1994) Psychometric theory McGraw-hill series. Psychology

[45] Glöckner-Rist A, Engberding M, Höcker A, Rist F. Prokrastinationsfragebogen für Studierende (PfS). [Procrastination Scale for Students.] In: Zusammenstellung sozialwissenschaftlicher Items und Skalen. [Summary of Items and Scales in Social Science.] GESIS, 2009. 10.6102/ZIS140

[46] Zhang S, Liu P, Feng T (2019). To do it now or later: the cognitive mechanisms and neural substrates underlying procrastination. Wiley Interdiscip Rev Cogn Sci.

[47] Paschke K, Arnaud N, Austermann MI, Thomasius R (2021). Risk factors for prospective increase in psychological stress during COVID-19 lockdown in a representative sample of adolescents and their parents. BJPsych open.

[48] R Core Team (2019). R: a language and environment for statistical computing.

[49] Brand M, Young KS, Laier C (2016). Integrating psychological and neurobiological considerations regarding the development and maintenance of specific Internet-use disorders: an interaction of person-affect-cognition-execution (I-PACE) model. Neurosci Biobehav Rev.

[50] Mahapatra A, Sharma P (2018). Association of Internet addiction and alexithymia—a scoping review. Addict Behav.

[51] Vine V, Aldao A (2014). Impaired emotional clarity and psychopathology: a transdiagnostic deficit with symptom-specific pathways through emotion regulation. J Soc Clin Psychol.

[52] Eisenberg N, Spinrad TL, Eggum ND (2010). Emotion-related self-regulation and its relation to children’s maladjustment. Annu Rev Clin Psychol.

[53] Chaku N, Hoyt LT (2019). Developmental trajectories of executive functioning and puberty in boys and girls. J Youth Adolesc.

[54] Fam JY (2018). Prevalence of internet gaming disorder in adolescents: a meta-analysis across three decades. Scand J Psychol.

[55] Bender PK, Reinholdt-Dunne ML, Esbjørn BH, Pons F (2012). Emotion dysregulation and anxiety in children and adolescents: gender differences. Personality Individ Differ.

[56] Sarıtaş-Atalar D, Gençöz T, Özen A (2015). Confirmatory factor analyses of the difficulties in emotion regulation scale (DERS) in a Turkish adolescent sample. Eur J Psychol Assess.

[57] Brix N, Ernst A, Lauridsen LLB (2019). Timing of puberty in boys and girls: a population-based study. Paediatr Perinat Epidemiol.

[58] Chaplin TM (2015). Gender and emotion expression: a developmental contextual perspective. Emotion Rev.

[59] Pascual A, Conejero S, Etxebarria I (2016). Coping strategies and emotion regulation in adolescents: adequacy and gender differences. Ansiedad y Estrés.

[60] Tsai J-K, Lu W-H, Hsiao RC (2020). Relationship between difficulty in emotion regulation and internet addiction in college students: a one-year prospective study. Int J Environ Res Public Health.

[61] González-Bueso V, Santamaría JJ, Fernández D (2018). Association between internet gaming disorder or pathological video-game use and comorbid psychopathology: a comprehensive review. Int J Environ Res Public Health.

[62] Young KS, Sandman CF, Craske MG (2019). Positive and negative emotion regulation in adolescence: links to anxiety and depression. Brain Sci.

[63] Niermann HCM, Scheres A (2014). The relation between procrastination and symptoms of attention-deficit hyperactivity disorder (ADHD) in undergraduate students: role of procrastination in ADHD-related symptoms. Int J Methods Psychiatr Res.

[64] Statista (2021) Share of households in Germany with internet access by 2020.

[65] Paschke K, Austermann MI, Thomasius R (2021). Assessing ICD-11 gaming disorder in adolescent gamers by parental ratings: development and validation of the gaming disorder scale for parents (GADIS-P). J Behav Addict.

[66] Taber KS (2018). The use of Cronbach’s alpha when developing and reporting research instruments in science education. Res Sci Educ.

[67] Lindenberg K, Kindt S, Szász-Janocha C (2022). Effectiveness of cognitive behavioral therapy-based intervention in preventing gaming disorder and unspecified internet use disorder in adolescents: a cluster randomized clinical trial. JAMA Netw Open.

[68] Torres-Rodríguez A, Griffiths MD, Carbonell X, Oberst U (2018). Internet gaming disorder in adolescence: psychological characteristics of a clinical sample. J Behav Addict.

[69] Moltrecht B, Deighton J, Patalay P, Edbrooke-Childs J (2021). Effectiveness of current psychological interventions to improve emotion regulation in youth: a meta-analysis. Eur Child Adolesc Psychiatry.

